# Investigation of Ifosfamide Toxicity Induces Common Upstream Regulator in Liver and Kidney

**DOI:** 10.3390/ijms222212201

**Published:** 2021-11-11

**Authors:** Hyoung-Yun Han, Mi-Sun Choi, Seokjoo Yoon, Je-Won Ko, Sang-Kyum Kim, Tae-Won Kim

**Affiliations:** 1 Department of Predictive Toxicology, Korea Institute of Toxicology, 141 Gajeong-ro, Yuseong-gu, Daejeon 34114, Korea; hanhy@kitox.re.kr (H.-Y.H.); mschoi@kitox.re.kr (M.-S.C.); sjyoon@kitox.re.kr (S.Y.); 2 Department of Human and Environmental Toxicology, University of Science and Technology, Daejeon 34113, Korea; 3 College of Pharmacy, Chungnam National University, 99 Daehak-ro, Yuseong-gu, Daejeon 34131, Korea; 4 College of Veterinary Medicine & Institute of Veterinary Science, Chungnam National University, 99 Daehak-ro, Yuseong-gu, Daejeon 34134, Korea; rheoda@cnu.ac.kr

**Keywords:** ifosfamide, hepatotoxicity, nephrotoxicity, intraperitoneal toxicity

## Abstract

Ifosfamide is an alkylating agent, a synthetic analogue of cyclophosphamide, used to treat various solid cancers. In this study, the toxicity of ifosfamide was evaluated using single-and multiple-dose intraperitoneal administration in rats under Good Laboratory Practice guidelines, and an additional microarray experiment was followed to support toxicological findings. A single dose of ifosfamide (50 mg/kg) did not induce any pathological changes. Meanwhile, severe renal toxicity was observed in the 7 and 28 days consecutively administered groups, with significant increases in blood urea nitrogen and creatinine levels. In the tox-list analysis, cholesterol synthesis-related genes were mostly affected in the liver and renal failure-related genes were affected in the kidney after ifosfamide administration. Moreover, interferon regulatory factor 7 was selected as the main upstream regulator that changed in both the liver and kidney, and was found to interact with other target genes, such as ubiquitin specific peptidase 18, radical S-adenosyl methionine domain containing 2, and interferon-stimulated gene 15, which was further confirmed by real-time RT-PCR analysis. In conclusion, we confirmed kidney-biased ifosfamide organ toxicity and identified identically altered genes in both the liver and kidney. Further comprehensive toxicogenomic studies are required to reveal the exact relationship between ifosfamide-induced genes and organ toxicity.

## 1. Introduction

Ifosfamide is an alkylating agent, a synthetic analogue of cyclophosphamide, used to treat various solid cancers, including sarcomas and lymphomas. Ifosfamide is a cell cycle nonspecific anticancer drug that interferes with DNA replication and RNA production [1]. Although ifosfamide is relatively well tolerated compared to other toxic alkylating agents, it is known to be associated with numerous life-threatening adverse effects that limit its clinical use [2]. The major side effects of ifosfamide include severe renal injury resulting from reactive toxic species from ifosfamide, including acute kidney injury, interstitial nephritis, hemorrhagic cystitis, and Fanconi syndrome [3]. In clinics, severe multiple organ toxicity was reported in patients who experienced early renal toxicity, and repeated high-dose ifosfamide-induced one organ failure that led to subsequent organ failure [4,5].

The toxic metabolite of ifosfamide, acrolein and chloroacetaldehyde, is a major responsible factor for ifosfamide organ toxicity. Previous studies have mainly focused on the factors affecting the conversion of ifosfamide to toxic metabolites, particularly cytochrome P450 (CYP) [6]. Relatively abundant CYP3A4 and CYP3A5 in kidney leads to toxic changes of ifosfamide and induces nephrotoxicity [7]. Acrolein is a highly reactive aldehyde that can activate intracellular reactive oxidative genes pathway and damage the cell organelle. Chloroacetaldehyde reported the suppression of the complex I activation in the mitochondrial respiratory chain that might lead to intracellular glutathione (GSH) depletion and cell death [8].

A multiple organ toxicological study is more helpful in understanding the systemic effects of drugs than targeting specific organs for toxicity studies. The liver and kidneys are major organs that participate in drug metabolism and excretion, and these major organs are predisposed to adverse drug reactions; organ toxicity often follows. Moreover, integrative toxicological evaluation can present various pathological findings that may help to understand and predict drug-induced toxicity in the system.

Many studies have used gene expression profiles to predict the potential negative effects of chemicals. However, most earlier studies focused on evaluating single-target organ toxicity, with only limited pathological symptoms [9]. Moreover, combined with microarray-based gene profiling, it will produce a comprehensive system-level understanding of toxicity that can prevent potential drug-related toxicity [10]. In this regard, integrative multi-organ toxicological approaches with gene expression profiling have been used in toxicity prediction model studies to overcome toxicity data derived from single organ toxicity evaluation, which is insufficient to understand the toxic mechanisms of drugs in the system [11].

In this study, we describe the toxicity of ifosfamide in the kidney and liver after acute and repeated exposure in Sprague–Dawley (SD) rats. Supportive gene expression profiling assays were performed to reveal the common target gene that was altered by ifosfamide in both the liver and kidney, suggesting an insight into integrative ifosfamide toxicity.

## 2. Results

### 2.1. Single Dose Toxicity Study

No toxicity was observed in the 12.5, 25, and 50 mg/kg ifosfamide groups—not death, toxic symptoms, body weight changes, or histopathological findings.

Meanwhile, in the hematology study, the relative counts of reticulocytes (RET) and absolute counts of neutrophils (NEUA) and lymphocytes (LYMA) decreased in a dose-dependent manner.

### 2.2. One-Week Repeated Dose Toxicity Study

Overall body weight decreased after 1 week of ifosfamide administration, and the mean values of body weight loss in the 100 mg/kg group were higher than those in the 75 mg/kg group (Table 1). Meanwhile, relative organ weight was gradually increased in ifosfamide-treated groups (Table 2).

One week of consecutive ifosfamide IP administration suppressed the hematology index, including RBC, HGB, HCT, MCV, and PLT, in a dose-dependent manner (Table 3). Moreover, BUN and CREA increased 1.2 times in the control group when compared to the ifosfamide group (Table 4). Kidney injury was further supported by a histopathological study.

**Figure 1:**
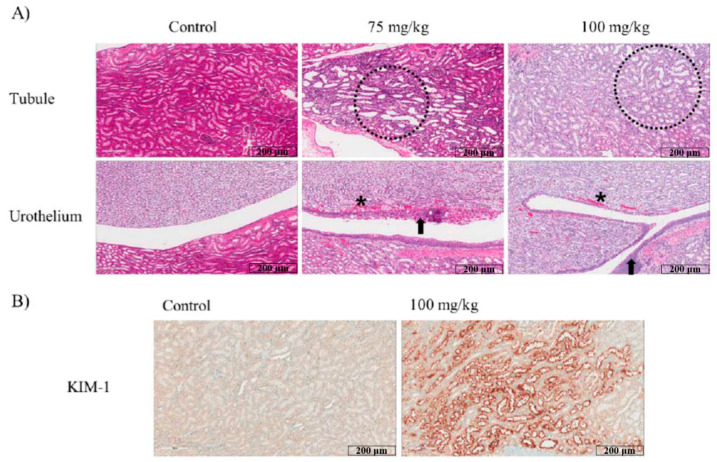
Histopathological changes in the kidney after 1 week consecutive ifosfamide (100 mg/kg) administration. (**A**) H&E-stained sections of kidney after 1 week of ifosfamide intraperitoneal injection. Dashed circle indicated tubular degeneration/dilatation. Arrow and asterisk indicated epithelial hyperplasia and focal hemorrhage/congestion, respectively. (**B**) Kidney section from 100 mg/kg group was found to have evidently increased KIM-1 expression in tubule area when compared to the control group.

No histopathological signs were observed in the livers of mice in the 75 and 100 mg/kg ifosfamide groups (Figure 1A). Meanwhile, the kidneys from the 75 and 100 mg/kg ifosfamide groups were found to have a minimal to slight grade of pathologic alteration, including tubular degeneration/dilatation (1 moderate in 100 mg/kg group), single cell necrosis, urothelium hyperplasia, and focal hemorrhage/congestion (Table 5). In addition, an evident increase in KIM-1 expression in the renal tubule area was observed in the kidney of the 100 mg/kg group (Figure 1B).

### 2.3. Four-Week Repeated Dose Toxicity Study

Mortality was observed in the 50 (5/10) and 70 (10/10) mg/kg-treated groups after 21 and 10 days of consecutive administration, respectively. Moreover, the decreased body weight with increased relative organ weight, liver, and kidney, in all ifosfamide-treated groups, supports 4-week repeated dose toxicity.

Hematological and serum biochemical analyses were performed on the surviving subjects. Several blood parameters, including RET, RBC, MONA, EOSA, and LUCA, were significantly decreased after 4 weeks of ifosfamide administration.

Compared with the control group, biochemical analyses showed statistical differences after 4 weeks of ifosfamide administration. For instance, the levels of AST and CK were significantly increased, and the A/G ratio, GLU, and GGT were decreased (*p* < 0.01) in the 50 mg/kg treated group, when compared with the control group.

### 2.4. Ifosfamide-Induced Gene Expression Analysis

The ifosfamide-induced DEGs were identified in the liver and kidney tissues (Table 6). For the liver, the differentially expressed genes included 2672 genes at 100 mg/kg bw/day, 1283 genes were upregulated, and 1389 genes were downregulated. In the kidneys, 401 genes were differentially expressed at 100 mg/kg bw/day—149 genes were upregulated, and 252 genes were downregulated. In both the liver and the kidney, the number of down regulated genes was greater than the number of up regulated genes. This might suggest that ifosfamide treatment appears to inhibit gene expression.

The biological function analysis results are listed in Table 7. The 1-week ifosfamide administration was found to alter genes related to organ development, mostly in the liver. In the kidney, it was confirmed that the genes related to immune cell localization were mostly affected.

**Figure 2:**
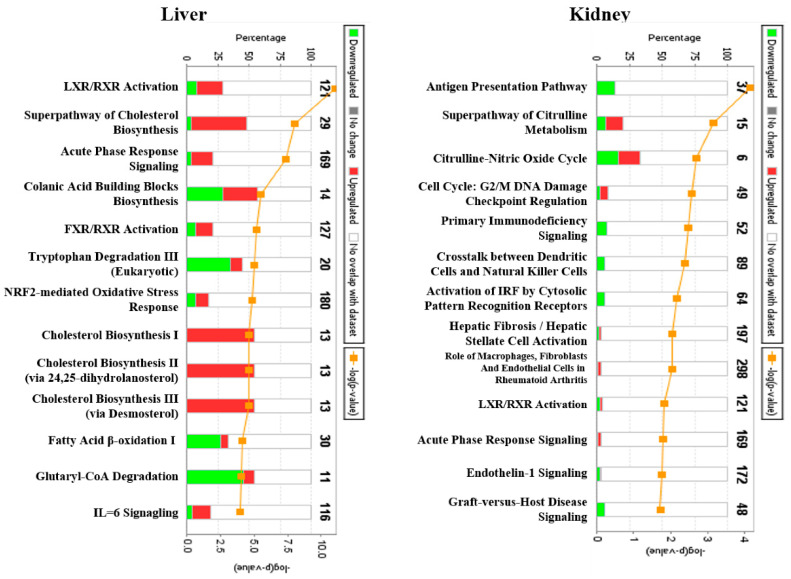
Top regulated canonical pathway analysis associated with 1-week consecutive ifosfamide (100 mg/kg) treatment. Each group of genes was selected based on a ≥1.5-fold change (9 < 0.05) cutoff using the GeneSpring program. The *p*-values were calculated based on Fisher’s exact test from the Ingenuity Pathways knowledge database.

In the canonical pathway analysis of DEGs, changes in cholesterol synthesis and antigen presentation pathway-related genes were evident in the liver and kidney tissues, respectively (Figure 2). Moreover, LXE/RXR and acute phase response signaling were the common top regulated canonical pathway in the liver and kidney, which were implicated in lipid homeostasis and inflammatory cytokine signaling, respectively.

In the liver tox-list analysis, cholesterol synthesis-related genes were mostly affected, followed by the positive acute phase response proteins and acute renal failure panel (Table 8). Furthermore, the kidney toxlist results revealed that the renal failure-related genes were generally affected by ifosfamide, including reversible glomerulonephritis biomarkers and acute renal failure panel.

**Figure 3:**
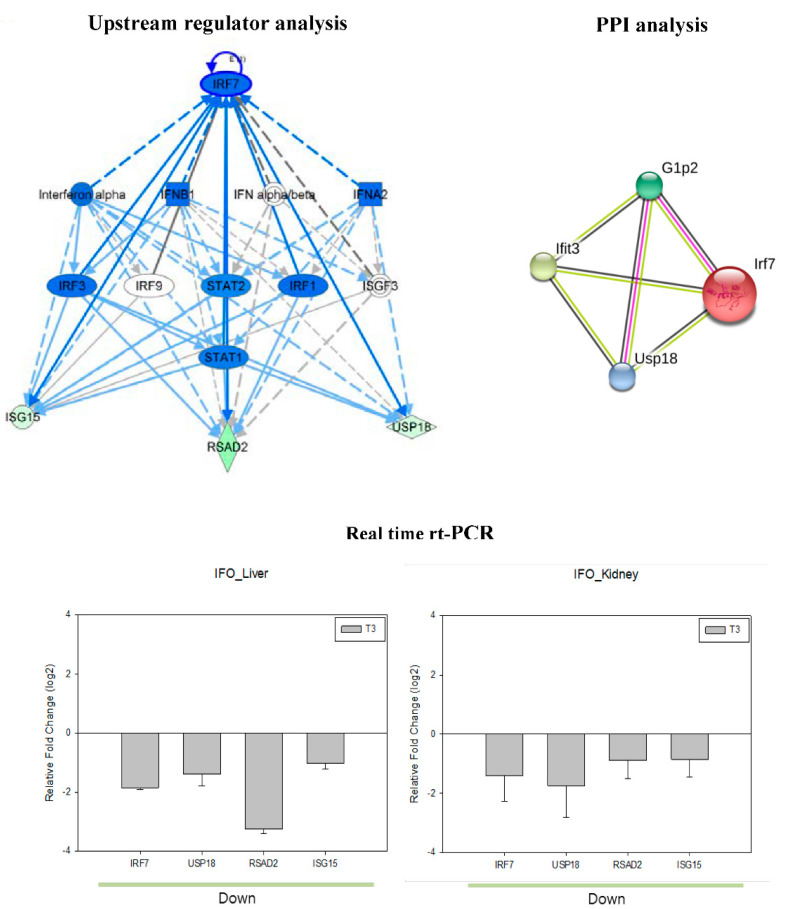
Upstream regulator and protein–protein interaction (PPI) network in ifosfamide toxicity. Upstream regulator analysis and PPI network analysis of the ifosfamide-induced DEGs and the expression of regulators in the liver and kidney were confirmed in the real-time rt-PCR analysis.

Based on the IPA knowledge base, a common upstream regulator regulates gene expression in the liver and kidney following ifosfamide treatment (Figure 3). IRF7 was the main regulator that changed in both the liver and kidney and was found to interact with other target genes such as USP18, RSAD2, and ISG15. Furthermore, real-time RT-PCR analysis confirmed the expression of IRF7, USP18, RSAD2, and ISG15. Although the extent of the changes in each gene was different between the liver and kidney, expression was suppressed in both liver and kidney tissues after 1 week of ifosfamide administration. The RSAD2 and USP18 were mostly altered gene by ifosfamide treatment in the liver and kidney, respectively.

## 3. Discussion

In this study, comprehensive ifosfamide toxicity in multiple organs was explored with different doses and exposure terms through the IP route in rats under the GLP standard. In clinics, ifosfamide can exert a bimodal antitumor action with cytotoxic and immunomodulatory effects combined with adoptive immunotherapy [12]. Ifosfamide is known to be mainly eliminated by the renal route and about 80% of the dose in the unchanged form, and nephrotoxicity is a commonly known side effect of ifosfamide, and metabolites such as chloroacetaldehyde and acrolein are responsible for its clinical use [13,14].

In the present study, a 1-week repeated toxicity study was suitable for exploring ifosfamide-derived organ toxicity. One week of ifosfamide administration suppressed the blood index in a dose-dependent manner, which might reflect typical ifosfamide-derived myelosuppression. Along with nephrotoxicity, several reports have indicated that myelosuppression is one of the major dose-limiting ifosfamide toxicities, and leucopenia is generally more severe than thrombocytopenia [15,16]. Ifosfamide nephrotoxicity can lead to Fanconi syndrome, in which there is an impairment of proximal tubule function resulting in irreversible damage, and the toxic metabolite acrolein provoke urotoxicity with hemorrhagic cystitis [17]. This is in accordance with the results of the present study. In the histopathological study, the kidneys and livers were examined to explore ifosfamide-derived pathological alterations. In both the 75 and 100 mg/kg ifosfamdie-administered groups, large areas of tubular degeneration and dilation were detected with utrothelium hyperplasia in the renal pelvis area. Furthermore, the expression of KIM-1, a renal proximal tubule injury molecule, supported the presence of ifosfamide-related nephrotoxicity. Meanwhile, only minimal periportal vacuolation was observed in the liver. Moreover, although the AST and ALT values decreased in a dose-dependent manner, the changes were within the reference range [18]. Deeper reasons for decreased liver enzyme levels will need to be further studied. It is generally accepted that ifosfamide is an alkylating agent that is infrequently associated with hepatotoxicity, and only a few cases have been reported to induce liver injury, especially when combined with other chemotherapeutic drugs such as doxorubicin [19].

Recently, notable progress in gene expression data in toxicology with high-throughput technologies has led to the generation of sufficiently large datasets in toxicity studies, and many attempts have been made to apply the gene profile to chemical toxicity prediction [10,20]. In this study, microarray analysis was conducted to analyze the transcriptome response induced by ifosfamide administration, and DEGs with a fold change ≥ 1.5 were selected as the target gene and the biological function, canonical pathway, and Tox list analysis were used to understand the function of ifosfamide-induced genes. An additional upstream regulator analysis was performed to evaluate the possibility of multi-organ toxicity in the liver and kidney.

In this study, DEGs changed 1.5-fold were selected, and the downregulated genes were slightly more dominant than the upregulated genes in both the liver (52%) and kidney (62%). The biological function analysis results indicated that ifosfamide changes the genes, with biological functions related to tissue development that are commonly observed in both the liver and kidney.

A previous study by Snouber et al. [21] reported ifosfamide treatment found to down regulate Nrf-2 mediated stress oxidative response pathways in the microfluidic culture [21]. Consistent with the previous studies, the NRF2-mediated oxidative stress response pathway was significantly modulated by top networks, which was confirmed by both the present canonical pathway and tox-list analysis. The present altered Nrf-2 mediated stress oxidative response pathways might be closely related to the ifosfamide toxic metabolite. Multiple ifosfamide toxic metabolites can react with GSH, potent antioxidant, to form conjugates at different sites along the pathway, and a decrease in the GSH level results in increased toxicity, which indicates the implication of oxidative stress in ifosfamide organ toxicity [8,22]. In this regards, mesna and N-acetylcysteine were used to alleviate ifosfamide organ toxicity. Mesna serves as a regional detoxificant by binding to ifosfamide toxic metabolite, mainly against acrolein, through a Michael addition to form a less harmful substance [23]. In addition, N-acetylcysteine reported to have antioxidant activity and improve GSH depletion under oxidative stress condition [24]. Further studies are needed to elucidate the exact relationship between ifosfamide antidote and pathway regulated under nephrotoxic condition. We can speculate that the ifosfamide toxicity amelioration by mesna or N-acetylcysteine might be followed by improved Nrf-2-mediated stress oxidative response pathways.

In this study, the inflammation-related signals were commonly upregulated in both the liver and kidney, which can be recognized by acute phase signaling in canonical pathway analysis. This result indicated the implication of inflammatory reaction in pathophysiology of ifosfamide organ toxicity in both tissues, which was further supported by the upstream regulator analysis. In the upstream regulator analysis, IRF7 and interferon (IFN) signal-related genes, including USP18, RSAD2, and ISG15, were selected as common upstream regulators in the liver and kidney. IRF7 is a lymphoid-specific factor, which is constitutively expressed in the immune cell cytoplasm, and can also be induced by type I interferon, virus infection, and external stimuli in various cells [25]. A previous study reported controversial pro- or anti-oncogenic properties of IRF7 in diverse tumor cells, and the changes in IRF7 expression were associated with DNA damage [26,27,28].

Addition to its important regulatory role in the type I IFN for antiviral functions, IRF7 reported to suppress inflammatory responses through TLR4 signaling pathway [29]. Moreover, Stout-Delgado et al. [30] reported that aging induced oxidative increment impairs IRF7 activity, whereas reduction of the stress by antioxidant agents improved IRF7 activity. In this study, ifosfamide administration suppressed the expression of upstream regulators, and the expression patterns were identical in both the liver and kidney. The present changes in IRF7 expression might relate to the inflammatory reaction and cytotoxic alkylating agent property of ifosfamide, which might suggest IRF7 as a promising target for ifosfamide organ toxicity.

In this study, we found a gene that was identically inhibited in both the liver and kidney after ifosfamide administration, but the interpretation of its toxicological significance was focused only on the kidney, which limits the current discovery. Moreover, further confirmatory studies related to the effect of the commercially available ifosfamide antidote on the currently altered gene might support the present findings.

In conclusion, a repeated toxicity study (1 week) of ifosfamide administration was found to have biased nephrotoxicity rather than hepatotoxicity. In addition, the present results provide evidence for an ifosfamide-induced hepatotoxicity and nephrotoxicity mechanism that involves IRF-7 inhibition. Further toxicogenomic studies of immune-related organs are required to elucidate the systemic toxicological mechanism of ifosfamide, and to support the relationship between gene and organ toxicity.

## 4. Materials and Methods

### 4.1. Animal Study

Eight-week-old male-specific pathogen-free Sprague–Dawley (SD) rats (n = 140) were obtained from Orient Bio Inc. (Seongnam, Korea). The animals were examined and acclimated to laboratory environmental conditions for a week before the experiment. All animals were housed in plastic cages under controlled laboratory conditions (temperature, 23 ± 3 °C; humidity, 55 ± 10%; and 12/12-h light/dark cycle) with laboratory chow and water ad libitum. The animal study was approved by the Institutional Animal Care and Use Committee of Korea Institute of Toxicology (Daejeon, Korea), and all procedures were performed in compliance with the Testing Guidelines for Safety Evaluation of Drugs from the Korea Food and Drug Administration. The animal study was divided into three sections: single dose (acute) and repeated dose (1 week and 4 weeks) consecutive administration toxicity study. Forty rats were randomly assigned to four groups (control, t1, t2, and t3) using the Path/Tox system (version. 4.2.2, Xybion Medical Systems Corporation, Lawrenceville, NJ, USA) in each toxicity study.

### 4.2. Toxicity Experiment

The toxicity study was divided into three sections: single dose (acute) and two repeated dose (consecutive dose of 1 week or 4 weeks) toxicity studies. For each toxicity study, rats were randomly assigned to adequate groups (control, dose 1, dose 2, and dose 3 for single and 4-week toxicity studies; control, dose 1, and dose 2 for 1-week toxicity study) by using the Path/Tox system (version. 4.2.2, Xybion Medical Systems Corporation, Lawrenceville, NJ, USA). In the toxicity study, ifosfamide was administered via the intraperitoneal (IP) route, and the control group received distilled water (DW). The ifosfamide dose used in the toxicity study was as follows: acute toxicity study (12.5, 25, and 50 mg/kg), 1-week toxicity study (75 and 100 mg/kg BW/day), and 4-week toxicity study (25, 50, and 70 mg/kg BW/day). The animals’ general clinical symptoms, body weight, and mortality were recorded daily during administration, and all animals were sacrificed 24 h after the last ifosfamide administration. Blood samples were collected and placed into microcentrifuge tubes and EDTA-K2 tubes for serum biochemistry and hematological analysis, respectively. Liver and kidney tissues were fixed in formaldehyde and subjected to histopathological analysis. Additional immunohistochemistry, microarray analysis, and real-time RT-PCR were performed on the liver and kidney tissues from the subacute toxicity study (100 mg/kg BW/day).

### 4.3. Hematological and Serum Biochemical Analysis

Standard hematological tests were performed using an ADVIA 120 hematology system (Bayer, Fernwald, Germany). The following indicators were used: white blood cell count (WBC), red blood cell count (RBC), hematocrit (HCT), hemoglobin concentration, mean corpuscular volume (MCV), mean corpuscular hemoglobin (MCH), platelet count (PLT), lymphocyte (LYM), monocyte (MON), neutrophil (NEU), basophil (BAS), eosinophil (EOS), and large unstained cells (LUC), and reticulocyte (RET) count.

The biochemistry assay was carried out using an automatic analyzer (TBA 120FR NEO; Toshiba Corp., Tokyo, Japan) with centrifuged serum. The main biochemical indicators were alanine aminotransferase (ALT), aspartate aminotransferase (AST), alkaline phosphatase (ALP), blood urine nitrogen (BUN), creatinine (CREA), glucose (GLU), total cholesterol (TCHO), albumin/globulin ratio (A/G), triglycerides (TG), total bilirubin (TB), gamma glutamyl transferase (GGT), phospholipids (PL), calcium, chloride, inorganic sodium, phosphorus, and potassium.

### 4.4. Histopathological and Immunohistochemistry Examination

Paraffin-embedded tissues were sectioned at 5-μm thickness, stained with hematoxylin and eosin (H&E), and examined microscopically.

The kidney tissue was subjected to immunohistochemistry. The deparaffinized and washed sections were preincubated with 10% goat serum to block nonspecific staining. The slides were then incubated overnight with the primary anti-KIM1 antibody (1:750; Santa Cruz, CA, USA). After the removal of primary antibodies, the sections were processed with VECTASTAIN Elite ABC HRP Kit (Vector Laboratories, Peterborough, UK), and KIM1 expression was examined under a light microscope.

### 4.5. Microarray Analysis and Protein–Protein Interaction (PPI) Analysis

The liver and kidney from the 1-week (100 mg/kg) group were homogenized, and the total RNA was extracted with an RNase mini kit (Qiagen). cDNA synthesis was achieved using a cDNA Synthesis Kit (Affymetrix, Affymetrix, Santa Clara, CA, USA) and subjected to microarray and PPI analyses.

Microarray analysis was performed using Affymetrix GeneChip Rat 230 2.0 with GeneChip Scanner 3000 (Affymetrix Santa Clara, CA, USA), and the results were processed using GeneSpring GX v13.0 analysis software (Agilent Technologies, Santa Clara, CA, USA). The differentially expressed genes (DEGs), which changed more than 1.5-fold after ifosfamide administration, were selected using one-way analysis of variance (ANOVA) with Tukey’s post hoc test. The biological functions and canonical pathways of the selected DEGs were analyzed using the Ingenuity Pathway Analysis software (IPA, ver. 9.0; Ingenuity Systems, Redwood City, CA, USA), and an upstream regulator analysis was performed to identify the upstream regulators that might be responsible for the DEGs derived from ifosfamide toxicity. Additional protein–protein interaction (PPI) network analysis of the DEGs was performed using STRING software (version 10), and the interaction was confirmed when the medium confidence score was over 0.4. Protein interaction is the approximate probability that a predicted link exists between two proteins in the Kyoto Encyclopedia of Genes and Genomes pathway.

### 4.6. Quantitative Real-Time RT-PCR Study

The expression of common regulator genes in the microarray study was confirmed using quantitative real-time RT-PCR. Gene-specific primers were obtained from Bioneer (Daejeon, Korea). The primer sequences were as follows: interferon regulatory factor 7 (IRF7): forward, 5-TGCTTGTCTAGCACCAATAG-3 and reverse, 5-CACAAGGTCCACTAGAGATG-3; ubiquitin specific peptidase 18 (USP18): forward, 5-CTGTAGTTTGTCTCCCAACA-3 and reverse 5-GAACTGATTACCTCCCACTG-3; radical S-adenosyl methionine domain containing 2 (RSAD2): forward, 5-ACCAATCATCAGAGGTTGAC-3 and reverse, 5-CTGCATGATTGTTCTTGGAC-3; interferon-stimulated gene 15 (ISG15): forward, 5-AAGTCTCCCAAGACCAATTC-3 and reverse, 5-CTACATTGGCTCTGGATAGG-3′.

Total RNA was reverse-transcribed to cDNA using SuperScript II (Invitrogen, Carlsbad, CA, USA) and oligo-dT primers, according to the manufacturer’s instructions. The mRNA expression levels of upstream regulator related genes were analyzed using the StepOnePlus Real-Time PCR System (Applied Biosystems, Carlsbad, CA, USA) with an SYBR Green master mix (Applied Biosystems), according to the manufacturer’s protocol. The 18S ribosomal RNA primer was used as an internal control, and the results were expressed as fold change relative to the normal control group.

### 4.7. Statistical Analysis

The data were statistically analyzed using multiple comparison methods. When Bartlett’s test showed no significant deviations from variance homogeneity, analysis of variance (ANOVA) was used to determine if any of the group means differed at the significance level of *p* < 0.05. Additionally, Dunnett’s test was used to determine differences in data between the control and treatment groups when the data were found to be significant from the ANOVA test. Furthermore, when significant deviations from variance homogeneity were observed from Bartlett’s test, a non-parametric comparison test, Kruskal–Wallis (H) test, was conducted to determine if any of the group means differed at the *p* < 0.05. When a significant difference was observed in the Kruskal–Wallis (H) test, the Dunn’s Rank Sum test was conducted to quantify the specific pairs of group data that were significantly different from the mean. Fisher’s exact test was conducted to compare pairs of data (including prevalence and percentage). The level of probability was set to 1 or 5%. Statistical analyses were performed by comparing the data from the different treatment groups with those of the control group using Path/Tox (version. 4.2.2, Xybion Medical Systems Corporation, Lawrenceville, NJ, USA).

## Figures and Tables

**Table 1:** Body weights results obtained on 1-week consecutive ifosfamide-administered rats.

Unit g	Body Weights		
	Control	75 mg/kg/day	100 mg/kg/day
Day 1	307.5 ± 6.3	312.1 ± 9.5	308.7 ± 11.7
Day 3	307.0 ± 7.8	297.8 ± 10.9	292.2 ± 13.7 *
Day 7	339.2 ± 13.1	307.9 ± 12.8 +	290.5 ± 18.6 +

+ Significant differences from control group *p* < 0.01. * Significant differences from control group *p* < 0.05.

**Table 2:** Absolute and relative organ weights obtained from 1-week consecutive ifosfamide-administered rats.

Unit (g)		1 Week after Dose		
		Control	75 mg/kg/day	100 mg/kg/day
Number Examined		10	10	10
Absolute weight	Liver	9.182 ± 0.8208	9.084 ± 0.8442	9.441 ± 0.8832
	Kidneys	2.557 ± 0.1997	2.661 ± 0.2088	2.705 ± 0.1281
Relative weight	Liver	2.963 ± 0.1932	3.030 ± 0.2370	3.228 ± 0.2301 *
	Kidneys	0.825 ± 0.0485	0.887 ± 0.0516 *	0.926 ± 0.0369 +

* Significant differences from control group *p* < 0.05. + Significant differences from control group *p* < 0.01.

**Table 3:** Hematological parameters obtained on 1-week consecutive ifosfamide-administered rats.

	Unit	1 Week Consecutive Dose		
		Control	75 mg/kg/day	100 mg/kg/day
WBC	×10^3^/µL	9.93 ± 2.35	0.58 ± 0.17 *	0.24 ± 0.15 +
RBC	×10^6^/µL	8.09 ± 0.39	7.28 ± 0.51 *	6.81 ± 0.95 +
HGB	g/dL	16.1 ± 0.50	15.0 ± 0.95 *	13.6 ± 1.81 +
HCT	%	50.7 ± 2.00	45.4 ± 3.30 +	41.2 ± 5.76 +
MCV	fL	62.7 ± 1.30	62.3 ± 1.42	60.6 ± 1.61 +
MCH	pg	20.0 ± 0.52	20.6 ± 0.55 *	20.0 ± 0.41
MCHC	g/dL	31.9 ± 0.43	33.1 ± 0.56 +	33.0 ± 0.44 +
PLT	10^3^/µL	1130 ± 131	21 ± 7.9 +	14 ± 6.2 +
RET	%	3.66 ± 0.53	0.06 ± 0.0 +	0.04 ± 0.0 +
RETA	10^9^/L	296 ± 47.9	4.2 ± 2.28 +	3.0 ± 1.56 +
NEU	%	12.2 ± 3.38	3.2 ± 1.36 +	4.4 ± 2.89 +
LYM	%	82.9 ± 3.79	93.4 ± 2.21 +	89.6 ± 5.16 +
EOS	%	1.1 ± 0.46	2.3 ± 1.11	3.7 ± 2.18 +
MON	%	2.7 ± 0.70	0.3 ± 0.26 +	0.5 ± 0.37 +
BAS	%	0.4 ± 0.12	0.2 ± 0.29	0.2 ± 0.37 *
LUC	%	0.6 ± 0.13	0.7 ± 0.46	1.6 ± 1.74
NEUA	10^3^/µL	1.23 ± 0.50	0.02 ± 0.01 +	0.01 ± 0.00 +

+ Significant differences from control group *p* < 0.01. * Significant differences from control group *p* < 0.05.

**Table 4:** Serum biochemical parameters obtained on 1-week consecutive ifosfamide-administered rats.

	Unit	1 Week Consecutive Dose		
		Control	75 mg/kg/day	100 mg/kg/day
GLU	mg/dL	91.0 ± 27.8	127.3 ± 24.9 +	163.6 ± 21.9 +
BUN	mg/dL	12.8 ± 1.45	16.8 ± 1.99 +	18.9 ± 6.68 +
CREA	mg/dL	0.43 ± 0.03	0.47 ± 0.03	0.51 ± 0.03 +
TP	g/dL	6.51 ± 0.29	6.20 ± 0.23	6.46 ± 0.39
ALB	g/dL	4.41 ± 0.16	4.21 ± 0.20 *	4.16 ± 0.21 *
A/G	ratio	2.13 ± 0.22	2.12 ± 0.23	1.82 ± 0.23 *
TCHO	mg/dL	65.1 ± 8.27	71.4 ± 12.6	69.6 ± 11.5
TG	mg/dL	42.2 ± 8.58	50.3 ± 10.8	49.2 ± 14.4
PL	mg/dL	97 ± 9.0	102 ± 14.0	107 ± 13.9
AST	IU/L	114.9 ± 9.09	60.0 ± 11.98 +	51.4 ± 8.59 +
ALT	IU/L	34.9 ± 3.45	22.9 ± 3.10 +	20.4 ± 4.44 +
TBIL	mg/dL	0.15 ± 0.00	0.17 ± 0.01 +	0.16 ± 0.02
ALP	IU/L	608.7 ± 75.9	379.3 ± 108.9 +	318.9 ± 111.3 +
CK	IU/L	505 ± 79.7	159 ± 30.2 +	126 ± 32.6 +
Ca	mg/dL	11.5 ± 0.36	13.0 ± 0.39 +	13.1 ± 0.41 +
IP	mg/dL	12.1 ± 0.66	11.2 ± 0.64 *	10.5 ± 0.96 +
Na	mmol/L	146 ± 0.8	146 ± 0.9	145 ± 1.3 *
K	mmol/L	8.69 ± 0.79	7.66 ± 1.23	7.93 ± 0.97
Cl	mmol/L	102 ± 1.0	104 ± 1.4	102 ± 2.5
GGT	IU/L	0.18 ± 0.29	0.15 ± 0.18	0.10 ± 0.23

+ Significant differences from control group *p* < 0.01. * Significant differences from control group *p* < 0.05.

**Table 5:** Histopathological findings on 1-week consecutive ifosfamide-administered rats.

	1 Week Consecutive Dose		
Dose	Control	75 mg/kg/day	100 mg/kg/day
Number Examined	10	10	10
**Kidneys**			
Basophilia tubule	2	3	2
Cast	1	4	2
Focal hemorrhage/congestion	0	2	2
Infiltration, inflammatory cell	2	0	0
Cystic tubules	0	0	1
Urothelium hyperplasia	0	2	2
Tubular degeneration/dilatation	0	7	4
Single cell necrosis	0	2	3
**Liver**			
Change, eosinophilic, hepatocyte, centrilobular	0	0	0
Congestion, centrilobular	0	0	0
Peripotal vacuolation	0	0	2
Fatty change, hepatocyte	0	0	0
Hypertrophy, hepatocyte, centrilobular	0	0	0
Infiltration, mononuclear, centrilobular	0	0	0
Infiltration, neutrophil, bile duct	0	0	0
Necrosis, hepatocyte, centrilobular	0	0	0
Mitosis	0	0	0
Single cell necrosis, centrilobular	0	0	0

**Table 6:** Differentially Expressed Genes (DEGs) 1 week after treating rats with ifosfamide at 100 mg/kg bw/day.

	X1.5 and 0.05			
	Dose	UP	DOWN	ALL
Liver	100 mg/kg	1283	1389	2672
Kidneys	100 mg/kg	149	252	401

**Table 7:** Biological function list of differentially expressed genes from ifosfamide-treated liver and kidney.

Liver	Kidney
Organismal Survival	Immune Cell Trafficking
Cardiovascular System Development and Function	Hematological System Development and Function
Organismal Development	Cardiovascular System Development and Function
Hematological System Development and Function	Tissue Morphology
Tissue Morphology	Tissue Development

**Table 8:** Tox-list analysis result associated with 1 week of consecutive ifosfamide (100 mg/kg) treatment.

Liver Tox list	Ratio	Kidney Tox List	Ratio
LXR/RXR Activation	2.85 × 10^−1^	Acute Renal Failure Panel (Rat)	1.13 × 10^−1^
Fatty Acid Metabolism	2.65 × 10^−1^	Reversible Glomerulonephritis Biomarker Panel (Rat)	1.85 × 10^−1^
Cholesterol Biosynthesis	6.88 × 10^−1^	Renal Necrosis/Cell Death	3.16 × 10^−2^
NRF2-mediated Oxidative Stress Response	1.75 × 10^−1^	Cardiac Hypertrophy	3.15 × 10^−2^
Increases Liver Damage	2.29 × 10^−1^	Cell Cycle: G2/M DNA Damage Checkpoint Regulation	7.69 × 10^−2^
FXR/RXR Activation	2.05 × 10^−1^	Renal Proximal Tubule Toxicity Biomarker Panel (Rat)	1.11 × 10^−1^
Positive Acute Phase Response Proteins	3.67 × 10^−1^	Genes Downregulated in Response to Chronic Renal Failure (Rat)	2 × 10^−1^
Cardiac Necrosis/Cell Death	1.59 × 10^−1^	LPS/IL-1 Mediated Inhibition of RXR Function	3.19 × 10^−2^
Acute Renal Failure Panel (Rat)	2.58 × 10^−1^	Recovery from Ischemic Acute Renal Failure (Rat)	1.43 × 10^−1^
LPS/IL-1 Mediated Inhibition of RXR Function	1.59 × 10^−1^	LXR/RXR Activation	4.07 × 10^−2^

## Data Availability

Data are contained within the article or Supplementary Materials.

## Supplementary Materials

The following are available online at https://www.mdpi.com/article/10.3390/ijms222212201/s1.
