# Supplementary material for: Investigation of Ifosfamide Toxicity Induces Common Upstream Regulator in Liver and Kidney
Source: Int J Mol Sci. 2021 Nov 11;22(22):12201. doi: 10.3390/ijms222212201 (PMC8617928; doi:10.3390/ijms222212201)
Supplement: Supplementary file 1 [file ijms-22-12201-s001.zip › ijms-1439873-supplementary.pdf]

Table S1. Hematological parameters of rats after single dose ifosfamide.

	Unit	1day after dose			
		V.C.<br>0.0mg/kg	T1<br>12.5 mg/kg	T2<br>25.0 mg/kg	T3<br>50.0 mg/kg
WBC	(x10^3/μL)	10.52±2.859	8.04±1.999*	6.80±1.430+	6.23±1.264+
RBC	(x10^6/μL)	8.19±0.984	8.18±0.475	8.11±0.381	8.34±0.574
HGB	(g/dL)	16.2±1.89	16.4±0.74	16.0±0.49	16.4±0.84
HCT	(%)	50.3±5.28	51.1±2.28	49.8±1.15	51.9±2.66
MCV	(fL)	61.6±1.88	62.6±1.67	61.5±2.39	62.3±2.32
MCH	(pg)	19.8±0.59	20.1±0.47	19.7±0.83	19.7±0.68
MCHC	(g/dL)	32.2±0.78	32.1±0.42	32.1±0.37	31.7±0.42
PLT	(10^3/μL)	1075±215.5	1195±130.1	1134±153.5	1179±161.1
RET	(%)	4.08±2.431	3.47±0.885	2.62±0.716	2.33±0.829*
RETA	(10^9/L)	314.4±115.39	280.7±60.63	211.4±54.11*	190.7±56.12+
NEU	(%)	16.4±7.70	8.1±4.14*	8.4±2.11	5.0±2.54+
LYM	(%)	78.1±7.93	86.3±4.84*	85.5±2.92	89.2±3.49+
EOS	(%)	0.9±0.29	0.8±0.26	1.1±0.38	0.8±0.42
MON	(%)	3.7±1.55	3.5±0.80	3.9±1.48	4.0±1.24
BAS	(%)	0.4±0.18	0.6±0.16	0.6±0.22	0.5±0.22
LUC	(%)	0.5±0.18	0.8±0.54	0.6±0.17	0.5±0.13
NEUA	(10^3/ μL)	1.77±1.174	0.65±0.355*	0.58±0.239*	0.31±0.162+
LYMA	(10^3/ μL)	8.16±2.127	6.94±1.823	5.80±1.116+	5.58±1.216+
MONA	(10^3/ μL)	0.40±0.227	0.28±0.091	0.28±0.140	0.25±0.065
EOSA	(10^3/ μL)	0.09±0.037	0.07±0.026	0.07±0.021	0.05±0.023+
BASA	(10^3/ μL)	0.04±0.020	0.04±0.012	0.04±0.014	0.03±0.014
LUCA	(10^3/ μL)	0.06±0.030	0.06±0.043	0.04±0.016	0.03±0.007+

* : Significant differences from control group (p<0.05)
\+ : Significant differences from control group (p<0.01)

Table S2. Serum biochemical parameters of rats after single dose ifosfamide.

	Unit	1day after dose			
		V.C.<br>0.0mg/kg	T1<br>12.5 mg/kg	T2<br>25.0 mg/kg	T3<br>50.0 mg/kg
GLU	(mg/dL)	110.9±16.91	100.2±17.56	125.4±26.16	130.7±49.25
BUN	(mg/dL)	11.4±1.63	12.2±1.71	13.2±2.12	12.7±1.77
CREA	(mg/dL)	0.47±0.032	0.47±0.042	0.48±0.037	0.49±0.043
TP	(g/dL)	6.34±0.152	6.47±0.202	6.51±0.167	6.59±0.184+
ALB	(g/dL)	4.26±0.088	4.31±0.101	4.32±0.102	4.40±0.080+
A/G	(ratio)	2.05±0.110	2.00±0.109	1.98±0.147	2.01±0.121
TCHO	(mg/dL)	53.8±12.04	53.3±12.32	57.0±7.70	60.8±9.93
TG	(mg/dL)	38.3±8.88	34.9±11.95	29.4±8.81	36.4±5.53
PL	(mg/dL)89±15.3	89±15.3	90±17.6	89±8.4	95±11.3
AST	(IU/L)	200.8±221.14	166.6±17.16*	155.3±21.02	134.0±27.12
ALT	(IU/L)	85.3±160.51	32.9±4.82	33.9±4.58	31.0±4.36
TBIL	(mg/dL)	0.108±0.0100	0.114±0.0125	0.107±0.0120	0.116±0.0107
ALP	(IU/L)	573.1±111.95	589.1±116.52	566.8±47.81	617.6±98.07
CK	(IU/L)	634±178.8	1077±161.5+	926±174.0*	762±321.8
Ca	(mg/dL)	11.08±0.323	11.20±0.316	11.01±0.362	11.09±0.338
IP	(mg/dL)	11.40±0.862	12.00±1.275	10.92±0.987	11.91±1.135
Na	(mmol/L)	146±1.3	147±1.4	146±1.3	147±2.0
K	(mmol/L)	9.13±1.021	8.83±1.129	8.94±1.170	8.99±1.242
Cl	(mmol/L)	105±1.6	104±1.6	104±1.3	105±1.3
GGT	(IU/L)	0.00±0.000	0.00±0.000	0.00±0.000	0.00±0.000

+ Significant differences from control group (p<0.01).

* Significant differences from control group (p<0.05).

Table S3. Urinalysis parameters of rats after single dose ifosfamide.

Unit (%)	1day dose				2day dose			
	V.C. 0.0mg/kg	T1 12.5 mg/kg	T2 25.0 mg/kg	T3 50.0 mg/kg	V.C. 0.0mg/kg	T1 12.5 mg/kg	T2 25.0 mg/kg	T3 50.0 mg/kg
UK	263.03±96.544	285.81±71.789	204.59±65.711	186.49±29.333	93.6±57.745	69.87±16.685	90.36±36.240	81.62±56.318
UCl	212±65.3	243±79.4	191±63.2	163±45.6	53±31.2	45±12.2	52±23.3	55±20.6
UNa	124±32.4	135±56.7	124±43.6	107±44.4	54±27.5	51±18.6	76±44.8	70±28.3

Table S4. Incidence of gross finding of rats after single dose ifosfamide.

Unit (g)	1day after dose			
	V.C.<br>0.0mg/kg	T1<br>12.5 mg/kg	T2<br>25.0 mg/kg	T3<br>50.0 mg/kg
Number Examined	10	10	10	10
Observed/No remarkable finding	10	10	10	10
No. Of Observations	10	10	10	10

Table S5. Absolute organ weights of rats after single dose ifosfamide.

Unit (g)	1day after dose			
	V.C.<br>0.0mg/kg	T1<br>12.5 mg/kg	T2<br>25.0 mg/kg	T3<br>50.0 mg/kg
Liver	9.133±1.0099	9.308±0.6444	8.731±0.7111	9.055±0.6346
Kidneys	2.505±0.2508	2.548±0.1782	2.561±0.2017	2.525±0.1776

Table 6. Relative organ weights of rats after single dose ifosfamide.

Unit (%)	1day after dose			
	V.C.<br>0.0mg/kg	T1<br>12.5 mg/kg	T2<br>25.0 mg/kg	T3<br>50.0 mg/kg
Liver	3.030±0.2701	3.096±0.2676	2.889±0.1713	3.033±0.1145
Kidneys	0.831±0.0642	0.847±0.0700	0.847±0.0475	0.846±0.0451

Table S7. Histopathological findings in rats after single dose ifosfamide.

	1day after dose			
Dose	V.C.<br>0.0mg/kg	T1<br>12.5 mg/kg	T2<br>25.0 mg/kg	T3<br>50.0 mg/kg
Number Examined	10	10	10	10
**Kidneys**				
Tubular basophilia	0	0	1	0
Dilated tubule	0	1	0	0
Cast	0	2	0	0
Interstitial fibrosis	0	1	0	0
Arteriopathy	0	0	1	0
**Liver**				
Infiltration, mononuclear	10	8	10	10
Focal necrosis	1	0	0	0
Arteriopathy	3	0	2	3
Extramedullary hemopoiesis	1	2	1	0
Vacuolated area	0	1	0	1
Subcapsular fibrosis	0	0	1	0

Table S8. Body weights and weight gains results obtained from 4week consecutive ifosfamide administered rats.

Unit (g)	Body weights				Weights gains			
	V.C. 0.0mg/kg	T1 25.0 mg/kg	T2 50.0 mg/kg	T3 70.0 mg/kg	V.C. 0.0mg/kg	T1 25.0 mg/kg	T2 50.0 mg/kg	T3 70.0 mg/kg
Day 1	171.6±3.07	170.8±4.54	171.5±5.20	172.5±3.74+				
Day7	221.3±3.73	207.3±6.51	192.1±10.84+	189.3±7.99+	49.6±3.57	36.5±4.30	20.6±8.51+	16.8±9.08+
Day14	276.5±8.59	245.1±10.82	191.8±22.57	-	104.9±8.17	74.3±8.17	20.2±20.2	-
Day21	284.8±10.99	231.7±20.13	169.5±19.73+	-	113.2±10.24	60.9±19.40+	-1.4±13.03+	-

+ : Significant differences from control group ($p<0.01$)

Table S9. Hematological parameters obtained from 4week consecutive ifosfamide administered rats.

	Unit	Dose		
		V.C.<br>0.0mg/kg	T1<br>25.0 mg/kg	T2<br>50.0 mg/kg
WBC	(x10^3/μL)	10.82±2.013	1.80±0.380	0.53±0.294
RBC	(x10^6/μL)	7.88±0.326	8.17±0.531	4.47±0.763+
HGB	(g/dL)	15.7±0.46	16.5±0.96	9.4±1.73
HCT	(%)	49.9±1.40	51.2±2.77	29.1±5.60
MCV	(fL)	63.4±2.02	62.8±1.25	61.3±4.14
MCH	(pg)	19.9±0.42	20.2±0.31	19.8±1.08
MCHC	(g/dL)	31.4±0.35	32.2±0.39+	32.3±0.73+
PLT	(10^3/μL)	13331±131.1	1124±213.0	734±529.3
RET	(%)	4.69±0.752	3.24±1.228	1.49±0.597+
RETA	(10^9/L)	368.0±49.98	265.2±97.61	73.1±34.46
NEU	(%)	10.7±3.47	25.8±12.11	35.0±7.24
LYM	(%)	83.1±3.70	56.5±11.23	46.9±11.51
EOS	(%)	0.6±0.22	1.7±0.95	1.8±1.51
MON	(%)	4.0±0.96	12.7±2.22	6.1±2.51
BAS	(%)	0.4±0.16	0.7±0.30	1.3±1.59
LUC	(%)	1.0±0.25	2.7±0.97	9.1±3.26
NEUA	(10^3/ μL)	1.14±0.358	0.47±0.248	0.17±0.078
LYMA	(10^3/ μL)	9.02±1.943	1.01±0.252	0.27±0.172
MONA	(10^3/ μL)	0.43±0.103	0.23±0.073+	0.04±0.030+
EOSA	(10^3/ μL)	0.07±0.026	0.03±0.024+	0.01±0.008+
BASA	(10^3/ μL)	0.05±0.018	0.01±0.006	0.00±0.004
LUCA	(10^3/ μL)	0.11±0.032	0.05±0.015	0.04±0.023+

\+ : Significant differences from control group (p<0.01)

Table S10. Hematological parameters obtained after 10 days of ifosfamide administration in rats.

	Unit	Dose
		T3<br>70.0 mg/kg
WBC	(x10^3/μL)	0.22±0.064
RBC	(x10^6/μL)	4.96±0.869
HGB	(g/dL)	9.7±1.75
HCT	(%)	29.5±5.94
MCV	(fL)	59.2±3.42
MCH	(pg)	19.5±0.59
MCHC	(g/dL)	92.9±1.06
PLT	(10^3/μL)	16±6.3
RET	(%)	0.10±0.019
RETA	(10^9/L)	4.8±1.14
NEU	(%)	8.0±7.73
LYM	(%)	86.5±10.38
EOS	(%)	2.0±1.47
MON	(%)	1.2±1.16
BAS	(%)	0.1±0.22
LUC	(%)	2.2±1.51
NEUA	(10^3/ μL)	0.02±0.023
LYMA	(10^3/ μL)	0.19±0.054
MONA	(10^3/ μL)	0.00±0.004
EOSA	(10^3/ μL)	0.00±0.005
BASA	(10^3/ μL)	0.00±0.000
LUCA	(10^3/ μL)	0.00±0.005

Table S11. Serum biochemical parameters obtained from 4week consecutive ifosfamide administered rats.

	Unit	DAY 21 Dose		
		V.C.<br>0.0mg/kg	T1<br>25.0 mg/kg	T2<br>50.0 mg/kg
GLU	(mg/dL)	82.8±12.37	89.9±25.64	133.9±23.92+
BUN	(mg/dL)	13.4±1.38	26.3±2.26	25.5±5.66
CREA	(mg/dL)	0.39±0.029	0.38±0.028	0.36±0.056
TP	(g/dL)	5.84±0.137	5.56±0.253	5.54±0.501
ALB	(g/dL)	3.96±0.077	3.83±0.186	3.53±0.263
A/G	(ratio)	2.10±0.128	2.23±0.119*	1.77±0.100+
TCHO	(mg/dL)	61.7±17.27	59.3±9.23	45.8±9.76
TG	(mg/dL)	23.7±8.77	18.6±3.24	13.3±1.06
PL	(mg/dL)89±1<br>5.3	82±16.2	88±7.9	77±14.8
AST	(IU/L)	125.6±22.62	89.0±13.98+	66.2±10.12+
ALT	(IU/L)	33.0±4.47	25.1±10.63	14.1±2.15
TBIL	(mg/dL)	0.129±0.0258	0.127±0.0217	0.111±0.0178
ALP	(IU/L)	850.3±160.65	411.2±78.16	443.8±51.39
CK	(IU/L)	644±102.4	417±143.0+	168±49.3+
Ca	(mg/dL)	10.78±0.505	10.94±0.339	10.68±1.024
IP	(mg/dL)	11.83±1.019	11.31±0.273	10.19±0.944
Na	(mmol/L)	147±4.7	148±1.1	148±1.9
K	(mmol/L)	9.27±1.776	8..28±1.443	8.42±1.949
Cl	(mmol/L)	102±3.6	104±1.0	108±2.3
GGT	(IU/L)	1.65±0.666	1.51±0.462	4.68±0.811+

* : Significant differences from control group (p<0.05)

+ : Significant differences from control group (p<0.01)

Table S12. Serum biochemical parameters obtained after 10 days of ifosfamide administration in rats.

	Unit	Day 10Dose
		T3<br>70.0 mg/kg
GLU	(mg/dL)	175.3±9.11
BUN	(mg/dL)	27.4±5.06
CREA	(mg/dL)	0.35±0.022
TP	(g/dL)	5.58±0.528
ALB	(g/dL)	3.71±0.228
A/G	(ratio)	2.02±0.224
TCHO	(mg/dL)	85.6±10.81
TG	(mg/dL)	24.6±7.86
PL	(mg/dL)89±15.3	118±22.7
AST	(IU/L)	56.9±3.18
ALT	(IU/L)	16.0±2.55
TBIL	(mg/dL)	0.207±0.0374
ALP	(IU/L)	440.7±147.50
CK	(IU/L)	111±31.3
Ca	(mg/dL)	10.83±0.430
IP	(mg/dL)	10.37±0.986
Na	(mmol/L)	142±4.2
K	(mmol/L)	6.81±1.103
Cl	(mmol/L)	100±7.9
GGT	(IU/L)	1.65±0.156

Table S13. Urinalysis parameters obtained from 4week consecutive ifosfamide administered rats.

Unit (%)	2day dose				8day dose			
	V.C. 0.0mg/kg	T1 25.0 mg/kg	T2 50.0 mg/kg	T3 70.0 mg/kg	V.C. 0.0mg/kg	T1 25.0 mg/kg	T2 50.0 mg/kg	T3 70.0 mg/kg
UK	89.36±50..964	93.26±33.634	108.49±44.930	61.89±31.995	83.61±45.929	77.06±36.531	77.61±18.199	55.10±16.648
UCl	72±38.2	71±21.9	78±27.6	52±19.7	59±26.2	60±27.1	51±11.2	43±9.7
UNa	58±27.1	67±24.4	68±19.6	46±18.1	45±16.3	48±16.5	39±11.2	39±12.1

Unit (%)	10 day dose	21day dose		
	T3 70.0 mg/kg	V.C. 0.0mg/kg	T1 25.0 mg/kg	T2 50.0 mg/kg
UK	84.73±32.571	71.98±24.784	73.71±37.931	100.40±94.174
UCl	56±27.0	40±10.9	46±17.2	44±37.4
UNa	62±19.2	40±10.9	46±21.1	39±28.5

Table S14. Absolute organ weights obtained from 4week consecutive ifosfamide administered rats.

Unit (g)	4week after dose			
	V.C.<br>0.0mg/kg	T1<br>25 mg/kg	T2<br>50.0 mg/kg	T3 (10days)<br>70.0 mg/kg
Number Examined	10	10	5	5
Liver	8.679±0.7366	7.376±0.7206	6.664±1.0905+	6.967±0.5784+
Kidneys	2.605±0.1265	2.219±0.2104	1.876±0.2064	1.652±0.1285+

+ : Significant differences from control group ($p<0.01$)

Table S15. Relative organ weights obtained from 4week consecutive ifosfamide administered rats.

Unit (%)	4week after dose			
	V.C.<br>0.0mg/kg	T1<br>25 mg/kg	T2<br>50.0 mg/kg	T3 (10 days)<br>70.0 mg/kg
Number Examined	10	10	5	5
Liver	3.053±0.2074	3.193±0.2664	3.934±0.3633+	4.491±0.5851+
Kidneys	0.917±0.0354	0.959±0.0569	1.112±0.0789+	1.060±0.0646+

+ : Significant differences from control group (p<0.01)

Table S16. Histopathological findings on 4week treating rats with ifosfamide at 25.0, 50.0 and 70.0 mg/kg bw/day.

	1day after dose			
Dose	V.C.<br>0.0mg/kg	T1<br>25 mg/kg	T2<br>50.0 mg/kg	T3 (10 days)<br>70.0 mg/kg
Number Examined	10	10	5	5
**Kidneys**				
Tubular basophilia	0	0	0	0
Cystic tubule	0	0	0	0
Tubular degeneration/regeneration	0	0	0	0
**Liver**	0	0	0	0
**Aorta**	0	0	0	0
